# Non-functioning pituitary adenomas and pregnancy: one-center experience and review of the literature

**DOI:** 10.20945/2359-3997000000232

**Published:** 2020-03-30

**Authors:** Josefina Rosmino, Julieta Tkatch, Maria Victoria Di Paolo, Silvia Berner, Sebastián Lescano, Mirtha Guitelman

**Affiliations:** 1 División Endocrinología Hospital General de Agudos Dr. Carlos G. Durand Buenos Aires Argentina División Endocrinología, Hospital General de Agudos Dr. Carlos G. Durand, Buenos Aires, Argentina.; 2 Unidad de Neurocirugía Hospital Santa Lucía Buenos Aires Argentina Unidad de Neurocirugía, Hospital Santa Lucía, Buenos Aires, Argentina; 3 Departamento de Neuroradiología División de Resonancia Magnética Hospital Juan A. Fernández –ARGUS Buenos Aires Argentina Departamento de Neuroradiología, División de Resonancia Magnética, Hospital Juan A. Fernández –ARGUS, Buenos Aires, Argentina

## Abstract

The usual clinical presentation of non-functioning pituitary adenoma (NFPA) consists of symptoms of mass effect and hypopituitarism. NFPA is a rare condition in young women and an uncommon complication during pregnancy. We present the outcome of three patients with NFPA during pregnancy. Case 1: a 38-year-old woman was referred at 32^nd^ week of spontaneous pregnancy because of diagnosis of a pituitary macroadenoma discovered in the context of progressive visual loss. Hormonal deficiency and hypersecretion were ruled out. Prolactin levels were high as expected. She developed diplopia and severe headache despite the use of dopamine agonists and corticosteroids, so pregnancy was interrupted at 34^th^ week. After an uncomplicated delivery of a healthy newborn, transsphenoidal surgery was performed. The pathology was consistent with a gonadotroph adenoma. She recovered visual field, and remained with normal pituitary function. Postsurgical tumor remnant increased in size during the follow-up. Case 2: a 34-year-old woman was referred due to secondary amenorrhea and galactorrhea. A macroadenoma with suprasellar extension was discovered. Transsphenoidal surgery confirmed a gonadotroph adenoma. Two years after surgery she had a normal pregnancy. Six years after surgery a small tumor recurrence occurred. Case 3: a 23-year-old woman was referred due to a microincidental pituitary adenoma. Laboratory testing was normal. No findings on physical examination. A wait and see approach was decided. Two years after diagnosis, the patient got pregnant without complications. Image remained stable. This article may contribute new cases and provides an extensive review of NFPA during pregnancy.

## INTRODUCTION

Fertility is commonly affected in patients with pituitary tumors. This is due to hormonal hypersecretion or mass effect, which causes destruction of gonadotrops or pituitary stalk compression, and leads to hyperprolactinemia and anovulation (1). During pregnancy, the pituitary gland experiences changes in anatomy and physiology. Its size may expand an average of 120% during normal pregnancy (2,3). The percentage of lactotrophs increases up to 40% in response to elevated maternal estrogen that stimulates prolactin secretion (2,3). However, symptoms such as blurred vision and headache resulting from physiologic pituitary enlargement are very rare (4). Prolactinomas are common in women of childbearing age. However, as they are associated with infertility, their diagnosis during pregnancy is uncommon. They are the most frequent pituitary tumor related to pregnancy. Tumor growth causing significant symptoms and requiring intervention has been reported to occur in 2.7% of patients with microadenomas, and in 4.8% and 22.9% of patients with macroadenomas with or without prior surgery or irradiation, respectively (5). Fertility may also be impaired in women with acromegaly, and in most of the over 100 cases published, patients have been diagnosed and operated on prior to pregnancy. The few cases of tumor growth during pregnancy were more frequent when the diagnosis was made during pregnancy (6-8). Nonfunctioning pituitary adenomas (NFPAs) are infrequent tumors in women of reproductive age, and few cases have been diagnosed and complicated during pregnancy. Recently, 28 cases of women with NFPA associated with pregnancies have been reported. Eight macroadenomas were diagnosed during pregnancy; 6 had signs and symptoms of tumor compression (9,10).

The aim of this study was to report three new cases of women with a diagnosis of NFPA in relation to pregnancy, one of them diagnosed and complicated during gestation, and to provide an extensive review of this topic.

## CASE 1

A 38-year-old woman was referred from the Ophthalmology Department at 32^nd^ week of spontaneous pregnancy for progressive visual loss during the last two months.

The patient reported three previous pregnancies, births by caesarean section without complications. She had regular menses before pregnancy, no headaches or visual defects. She had no relevant past medical or surgical history. No findings on physical examination.

The onset of symptoms occurred at 24^th^ week with decreased visual acuity in the right eye. High-dose prednisone was administered for one week for suspicion of optic neuritis, with no improvement of symptoms. Fifteen days later, the patient also developed decreased visual acuity in the contralateral eye.

**Figure 1:**
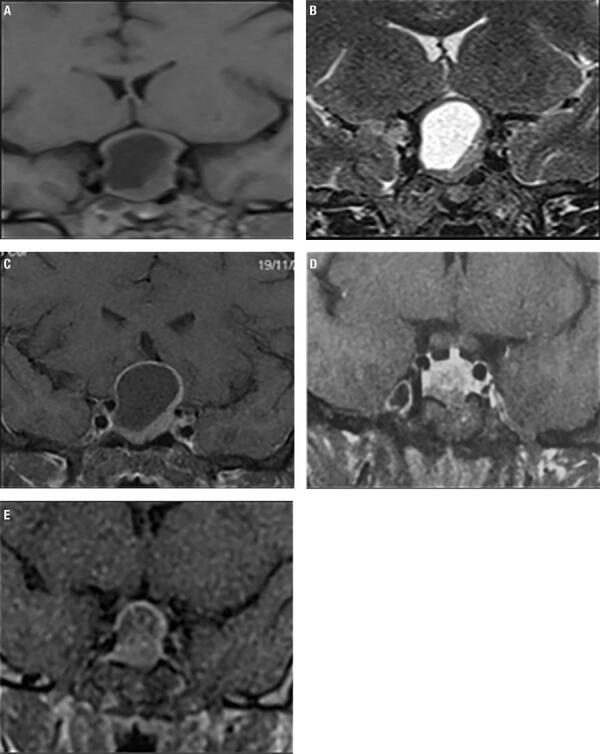
Case 1 Pituitary MRI without gadolinium during pregnancy (A-B) and postpartum/postsurgery MRI with gadolinium (C-D-E): Coronal view of a large pituitary mass, hypointense on T1-weighted images (A), and hyperintense on T2-weighted images, with suprasellar extension and compression of the optic chiasm (B). Coronal view of a stable post-partum image after gadolinium-enhancement (C). Post-surgery pituitary MRI with gadolinium: coronal view of a small tumor remnant, T1-weighted images (D). Coronal T1-weighted image shows tumor re-expansion after 4 years of surgery (E).

MRI without gadolinium, performed at 32 weeks of gestation, showed a voluminous pituitary lesion with suprasellar extension and compression of the optic chiasm. These findings were suggestive of a pituitary macroadenoma with cystic component (Figure 1A-B).

A new ophthalmologic examination revealed blurred vision on the right eye, and 8/10 visual acuity on the left eye. The computerized visual field showed left hemianopsia and could not be performed in the right eye due to amaurosis. Optic nerve atrophy had not been evaluated because the patient did not have the possibility of undergoing optical coherence tomography (OCT).

Laboratory testing was normal for thyroid and GH axis. PRL levels were 274 ng/mL, as expected for pregnancy at week 32. Cortisol was not evaluated because the patient was receiving glucocorticoids. There were no symptoms of diabetes insipidus; serum electrolytes and serum/urine osmolality tests were within normal limits. There were no signs or symptoms of excess GH or cortisol.

As in Argentina Bromocriptine is not available, Cabergoline 0,5 mg/week was initiated and corticosteroids were restarted, but 72 hours later, she developed diplopia, paresthesia in upper and lower limbs and severe headache. Cesarean section was performed at week 34 to interrupt pregnancy. She delivered a healthy newborn with no complications. Six days after delivery, a new MRI was performed with intravenous contrast, showing a voluminous sellar lesion with suprasellar extension, revealing no changes from the previous image obtained during pregnancy (Figure 1C). A brain CT scan showed no calcifications suggestive of craniopharyngioma.

**Figure 2:**
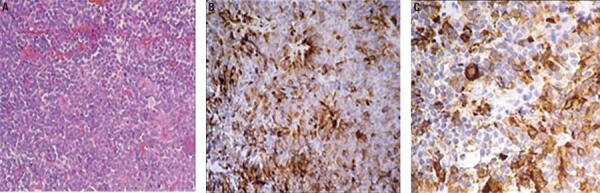
Case 1 – Histologic pictures of the removed Gonadotroph adenoma: (A) Haematoxylin – eosin staining (x400), (B) Immunostaining for LH (x400), (C) Immunostaining for FSH (x400).

Cabergoline was continued with the aim of inhibiting breastfeeding. The patient and her baby were discharged and referred to the Neurosurgery unit. Transsphenoidal surgery was performed in a center with experience in pituitary surgery. The immunohistochemistry was positive for FSH and LH, and negative for prolactin, ACTH, and GH; Ki67 was 2.8% (Figure 2). After surgery, the patient fully recovered visual field and menstrual cycles, and remained with normal pituitary function*. *Cabergoline was suspended. Three months after surgery, prolactin increased to 30-60 ng/mL, and MRI revealed a small residual tumor (Figure 1C) which presented re-growth after a four year follow up (Figure 1E).

## CASE 2

A 33-year-old woman was referred to our Unit due to secondary amenorrhea and galactorrhea, without headaches, or visual disturbances. As obstetric history, she had two previous pregnancies, one ended in miscarriage and the other in an uncomplicated cesarean with a healthy newborn.

**Figure 3:**
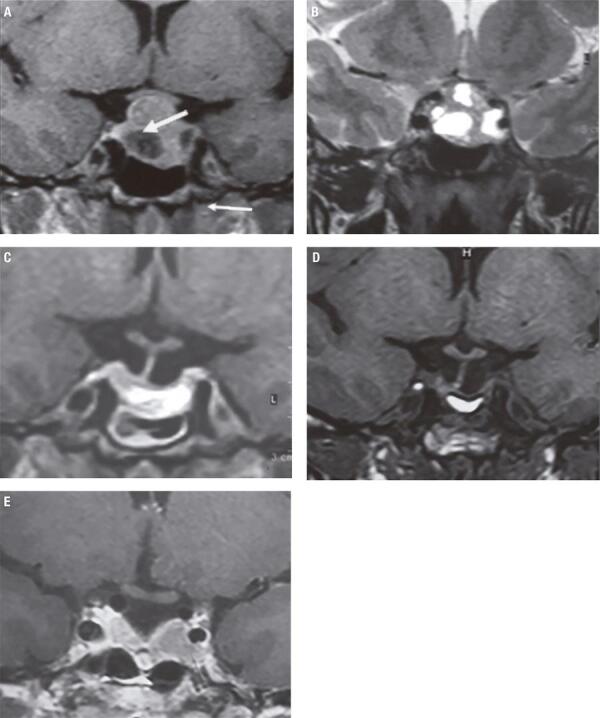
Case 2 – Pre-surgery pituitary MRI (A-B): Coronal view of a heterogeneous lesion with solid and cystic areas. T1 after gadolinium administration (A) and T2-weighted images (B) showed left cavernous sinus and suprasellar extension with compression of the optic chiasm. Post-surgery MRI (C-E): Coronal T1-weighted image showed partial empty sella and spontaneously hyperintense filling material. No visible tumor remnant (C) MRI performed after 6 months of a non-complicated pregnancy; the lesion remained stable (D). MRI six years after pregnancy. Contrast-enhanced coronal T1-weighted image shows a small tumor recurrence close to the left cavernous sinus (E).

Laboratory testing revealed hyperprolactinemia with levels lower than 100 ng/mL; no GH or ACTH excess were found, and pituitary function was normal. MRI demonstrated a pituitary macroadenoma with suprasellar extension and compression of the optic chiasm (Figure 3A-B); computerized visual field showed a small right superior temporal defect. In spite of suspected NFPA, she was initially treated with DA. After one year under unsuccessful treatment with cabergoline, she underwent transsphenoidal surgery, which provided the diagnosis of a Gonadotroph adenoma. During follow-up, the patient recovered visual field and menstrual cycles, maintaining normal pituitary function. No pituitary tumor remnant was seen after surgery, and there were no tumor recurrences during the two-year follow up (Figure 3C-D).

The patient spontaneously became pregnant two years after surgery. No neuro-ophthalmologic complications during pregnancy were reported, and a healthy baby was born in term. MRI after 6 months of delivery remained stable (Figure 3), without tumor re-expansion in the following 4 years. She is currently 39 years old, remains asymptomatic, but the MRI scan showed a small tumor recurrence 6 years after surgery (Figure 3E).

## CASE 3

A 23 -year-old woman was referred to our Unit due to a pituitary incidental microadenoma of 7x 6 mm in size. MRI was requested for headache complaints. She had regular menses and no visual defects. No relevant past medical or surgical history or findings on physical examination.

Laboratory testing was normal for pituitary function, suggesting NFPA.

The therapeutic decision was to wait and see, with clinical follow-up and tumor growth monitoring.

One year after diagnosis, the patient got pregnant; during pregnancy, she remained asymptomatic without neuro-ophthalmologic complication. She delivered a full-term healthy newborn. During follow-up and after breastfeeding, the patient recovered regular menstrual cycles, and remained with normal pituitary function. MRI did not show any changes from baseline. She was lost to follow-up two years after delivery.

## DISCUSSION

According to the new classification system using transcription factors, gonadotroph adenomas comprise 73% of NFPA (11). The latter represents 30%-40% of all surgically treated pituitary adenomas (12).

NFPAs constitute the most frequent form of macroadenoma and are mostly seen in postmenopausal women and men over 50 years (13). These tumors have a prevalence of 50-60 cases per million inhabitants and an incidence of 4-5 cases per million inhabitants per year (14). Although they produce gonadotropins and/or their alpha/beta subunits, there is usually no clinical evidence of hormone hypersecretion (13,15).

NFPAs are difficult to recognize clinically until they are large enough to cause symptoms due to a mass effect (15,16). Almost 50% are detected as an incidental finding when MRI is done for other reasons (17-19). Hyperprolactinemia was found in 45.2% of a series of 66 macroadenomas with NFPA, with a mean value of 65.6 ng/mL (20). At the time of diagnosis, 50%-60% of patients with macroadenomas present visual impairment caused by suprasellar extension. The most common symptoms are bitemporal hemianopsia or visual field disturbances and 30%-40% develop headache (18).

Histologically, this is a heterogeneous group of tumors currently classified according to their pituitary cell lineage. NFPA can also be silent, expressing but not secreting other anterior pituitary hormones, with the most common being silent ACTH adenomas. Most tumors that were previously classified as “null cell” adenomas based on negative hormone immune-staining can now be re-classified based on transcription factors analysis (11,21,22).

In case of suspicion, a computerized visual field, pituitary MRI and testing for hypopituitarism should be performed (13,15). The initial treatment of these tumors consists in transsphenoidal resection when possible (23,24). Stereotactic radiotherapy is used to treat and prevent recurrences as well as to reduce the size of postsurgical remnants (13,15). Pharmacological therapy with somatostatin receptor ligands and/or dopamine agonists has not been demonstrated to be effective in reducing tumor size (13,16,25). In a recent publication, DAs were considered in patients with NFPA with incompletely resected tumors, as the authors demonstrated that DA therapy may prevent residual tumor enlargement in over 85% of patients with NFPA (26).

Gonadotroph adenomas are uncommon tumors in women of reproductive age and in pregnancy because fertility is usually affected due to hypogonadotrophic hypogonadism, mostly secondary to mass effect.

When hyperprolactinemia is present, a well-defined cutoff value will be of great help in the clinical management of patients with a large pituitary mass. Karavitaki and cols. found prolactin levels of less than 100 ng/mL in 226 patients harboring null cell or gonadotroph macroadenomas and not receiving drugs known to elevate prolactin levels (27). In addition, the Endocrine Society guidelines for the diagnosis and treatment of hyperprolactinemia 2011 suggested that prolactin levels < 94 ng/mL can distinguish between macroprolactinoma and NFPA (28).

However, diagnosis of NFPA may occur during pregnancy (Table 1), since the enlargement of the pituitary gland may reveal the emergence of symptoms related to mass effect, as visual field defect or headache, in patients with previous undiscovered PT (29).

**Table 1:** Published data of patients with NFPA diagnosed and complicated during pregnancy

	NFPA (Non Functioning Pituitary Adenoma)	Gestational time at diagnosis	Presenting symptoms	MRI pretreatment	Treatment during pregnancy	MRI post initial treatment	Evolution	Laboratory findings	Gestacional outcome	Treatment after pregnancy
Masding, 1996^35^	1	2^nd^ trimester	Vision loss in the left eye	Pituitary enlargement with compression of the optic chiasm	DA^1 ^(bromocriptine up to maximum dose of 15 mg/day)until postpartum	Decompression of the optic chiasm	After DA suspension, visual symptoms reappeared	PRL^4 ^increased (100 mU/L). No other findings	Delivery without complications	TSS^5 ^(confirm NFPA)
de Heide, 2004^37^	1	2^nd^ trimester	Pituitary apoplexy	Pituitary enlargement with hyperintense image on t1	Symptomatic treatment (desmopressin and glucocorticoids)	Regression of pituitary mass	Panhypopituitarism	PRL^4^ increased (912 mU/I) Adrenal insufficiency. Central hypothyroidism. ID^2^	Delivery without complications	No
Hye-Ran, Lee 2014^4^	1	3^rd^ trimester	Diplopia and left ptosis	Macroadenoma 1,5 x 1,3 x 1cm with suprasellar extension and compression of the optic chiasm	DA^1^ (third trimester, bromocriptine 2.5 mg three times daily) until postpartum	Decreased lesion size 1,3 x 1,1 x 0,7cm	Diplopia and neurological disorders disappeared	PRL^4^ increased (176.3 ng/mL)	Vacum extraction delivery	No
Lambert, 2017^9^	4	3^rd^ trimester	Focal neurology	ND^3^	ND^3^	ND^3^	ND^3^	ND^3^	Cesarean section	No
		2^nd^ trimester	Visual loss	ND^3^	TSS^5^ (2^nd^ trimester)	ND^3^	ND^3^	ND^3^	Cesarean section	ND^3^
		3^rd^ trimester	Absence episodes	ND^3^	DA^1^ (3^rd^ trimester)	ND^3^	ND^3^	ND^3^	Cesarean section	ND^3^
		ND^3^	ND^3^	ND^3^	ND^3^	ND^3^	ND^3^	ND^3^	ND	ND^3^
Karaka, 2018^10^	1	1^st^ trimester	ND^3^	ND^3^	No	ND^3^	ND^3^	ND^3^	Miscarriage (at 8 weeks)	ND^3^
Rosmino, 2019	1	2^nd^ trimester	Decreased visual acuity in the right eye	Pituitary lesion with suprasellar extension and compression of the optic chiasm	DA^1^ (3^rd^ trimester) suspended after surgery	No changes	Diplopia and worsening of symptoms	PRL^4^ 274 ng/mL. No other findings	Cesarean section (week 34)	TSS^5^ (confirm NFPA)

DA^1^: dopamine agonist; ID^2^: insipidus diabetes; ND^3^: no data; PRL^4^: prolactin; TSS^5^: transsphenoidal surgery.

We reported 3 cases of patients with NFPA related to pregnancy, one of them diagnosed and complicated during pregnancy without previous evidence of a pituitary adenoma, so that diagnosis during the course of gestation was difficult.

Among differential diagnoses, a complicated undiagnosed *macroprolactinoma *during pregnancy is uncommon; most patients are diagnosed previously due to symptoms related to PRL excess. Levels of PRL do not help during pregnancy (30). Our first case had no previous history of gonadal dysfunction and presented high PRL levels, consistent with the physiologic increase during pregnancy. The possibility of a physiologic enlargement during pregnancy due to lactotroph hyperplasia is unlikely because of the great visual field disturbance, and the fact of postpartum worsening (4).

The diagnosis of *GH-producing adenoma* was not suspected due to the lack of typical clinical features; however, normal IGF1 levels were only confirmed after childbirth, since both the influence of high estrogen levels and placental GH are associated with variations in GH and IGF1 levels during gestation (8,31).

We ruled out the possibility of an apoplexy in this patient based on the clinical presentation and MRI scan. Moreover, no elements of hemorrhage were found in the surgical piece on pathological examination. We observed a pituitary mass predominantly hypointense on T1- and hyperintense on T2-weighted images, related to a predominantly cystic pituitary adenoma. In this patient, the MRI was performed fifteen days after the onset of symptoms; in case of pituitary apoplexy we should have observed a predominantly hyperintense T1-weighted image (32).

*Lymphocytic hypophysitis* (LH) is the most common among inflammatory processes affecting the pituitary gland, especially in the last semester of pregnancy and in the post-partum period (33). The lack of response to high doses of corticosteroids, the non-typical MRI image, led us away from the diagnosis of hypophysitis in our first case. Finally, isolated reports exist on patients with *craniopharyngioma *who became pregnant after surgical treatment and irradiation (34). In our first patient, a brain CT was performed and no characteristic calcifications of this type of tumor were found, although the lesion presented with cystic changes. Craniopharyngioma is usually associated with gonadal deficiency and diabetes insipidus, features which were not present in the current case.

Pregnancy has been rarely associated with increased tumor size of clinically non-functioning pituitary adenomas.

Lee and cols. reported a case of a 39-year-old woman presenting with diplopia and blurred vision at 33 weeks of gestation. The MRI revealed a pituitary macroadenoma with suprasellar extension suspected of being a NFPA. Despite treatment with bromocriptine, there was no significant visual field improvement during pregnancy. After 1 month of delivery, diplopia disappeared and visual examination was normal. Five months later, the MRI showed a significant decrease in tumor size. Nevertheless, diagnosis remained uncertain because of the lack of surgical intervention and histology (4).

Another publication reported the case of a 29-year-old woman with a history of oligomenorrhea and anovulation, in whom hyperprolactinemia had been ruled out. During pregnancy, the patient experienced loss of vision and an MRI scan showed a sellar mass with optic chiasm involvement. The patient received bromocriptine with size reduction of the sellar mass and visual field recovery. Postpartum, the visual field defect returned and she then underwent transsphenoidal decompression of the tumor. On histological examination, it proved to be a pituitary adenoma in which only about 5% of cells stained for PRL and GH. Based on this data, the authors considered it was a NFPA (35).

Murata and cols. reported an exceptional case of a 29-year-old woman with a gonadotroph microadenoma with ovarian hyperstimulation who responded to bromocriptine and became pregnant during treatment. The course of pregnancy was uneventful, but both ovaries gradually became enlarged in spite of the continued bromocriptine treatment until the 34th week of gestation. The tumor was successfully removed after 3 years of an uncomplicated delivery. Immunohistochemical analysis of the tumor showed strong positive reactivity for FSH, and weak for PRL (36).

De Heide and cols. described a 26-year-old woman who was admitted at 23 weeks of gestation with a sudden onset of severe headache, vomiting, disturbed consciousness and photophobia. MRI showed a pituitary macroadenoma with signs of apoplexy. Complete hypopituitarism was present at the time of diagnosis, and after treatment with glucocorticoids, she developed diabetes insipidus, together with clinical improvement of neuroophthalmologic symptoms. A repeat MRI scan eight weeks later showed regression of the pituitary mass with a small remnant in the left cavernous sinus. Delivery after 38 weeks of pregnancy was uneventful and a healthy baby was born. This case was considered by the authors as an apoplexy of a NFPA during pregnancy, an extremely rare situation (37).

Recently, two series with a larger number of women with NFPA and pregnancy have been published (9,10).

Lambert and cols. reported 16 cases of NFPA in pregnant women (9). Four were diagnosed during pregnancy; three of them with symptoms of tumor expansion. One out of 12 women diagnosed before conception also experienced symptomatic tumor expansion. This patient had not been previously operated on, or received any treatment with DA. Of the four women who presented symptomatic tumor expansion during pregnancy, two were in the second trimester and two in the third. The symptoms described were focal neurology, headaches with pituitary apoplexy, absence episodes and visual loss. Only the patient with visual loss required surgery; of the remaining three, two received cabergoline, and no data is available on the fourth. There is no information about the histological type of NFPA in this cohort of patients or about the follow-up of the patients and newborn after delivery. There is no data either about surgery or radiotherapy for the women who were diagnosed prior to pregnancy. This series also included 49 women with macroprolactinomas, of which all but one were diagnosed prior to pregnancy. The authors concluded that NFPA occurs in pregnancy more commonly than previously thought and it can present de novo symptoms of tumor expansion during pregnancy.

The second new study from Karaca and cols. described 8 cases of NFPA (4 macro-, 4 microadenomas) during pregnancy (10). Only one macroadenoma was diagnosed during the first trimester, which ended with miscarriage at 8 weeks of gestation. This woman was also treated with salazopyrin and plaquenil for systemic lupus erythematosus. None of these reported cases had signs or symptoms of tumor compression. Pregnancy occurred spontaneously in 7 patients, and with ovulation induction in one patient who had secondary hypogonadism. One of the patients had TSH deficiency before and during pregnancy. One of the patients was on cabergoline at the time of conception and throughout gestation since she had an invasive adenoma, but did not develop compressive signs during pregnancy. Two cases ended with miscarriage, one previously described, and other patient was on sertraline for depression and levothyroxine for primary hypothyroidism. From 6 live births, one was macrosomic and no other problems were detected. As with the previous series, no information is given about the histological type of NFPA.

Of the 28 published cases of women with NFPA associated with pregnancy, 8 (28.5%) were diagnosed during pregnancy (Table 1), which is a high frequency compared to that shown in functioning adenomas (9,10,30). Moreover, 6 out of these eight cases presented with neuro-ophtalmological complication. Thus, NFPAs are being more recognized in relation to pregnancy than before. However, in most of the published studies, there is no information on the histological type, although it is presumed that most are gonadotropinomas.

This study adds three new cases of NFPA related to pregnancy, two histologically confirmed as gonadotroph adenomas, and one of them diagnosed and complicated during the course of pregnancy. The limitation of this study is the low number of patients in this series, which is justified by the limited association of NFPA with pregnancy compared with functioning adenomas.

The strength of this study is that it addresses a topic not so widely studied and that it encourages endocrinologists to publish their own series or cases.
